# Impact of Trichiasis Surgery on Quality of Life: A Longitudinal Study in Ethiopia

**DOI:** 10.1371/journal.pntd.0004627

**Published:** 2016-04-14

**Authors:** Esmael Habtamu, Tariku Wondie, Sintayehu Aweke, Zerihun Tadesse, Mulat Zerihun, Aderajew Mohammed, Zebideru Zewudie, Kelly Callahan, Paul M. Emerson, Robin L. Bailey, David C. W. Mabey, Saul N. Rajak, Hannah Kuper, Sarah Polack, Helen A. Weiss, Matthew J. Burton

**Affiliations:** 1 International Centre for Eye Health, London School of Hygiene & Tropical Medicine, London, United Kingdom; 2 The Carter Center, Addis Ababa, Ethiopia; 3 Amhara Regional Health Bureau, Bahirdar, Ethiopia; 4 The Carter Center, Atlanta, Georgia, United States of America; 5 International Trachoma Initiative, Atlanta, Georgia, United States of America; 6 Clinical Research Department, London School of Hygiene & Tropical Medicine, London, United Kingdom; 7 International Centre for Evidence in Disability, London School of Hygiene & Tropical Medicine, London, United Kingdom; 8 MRC Tropical Epidemiology Group, London School of Hygiene & Tropical Medicine, London, United Kingdom; University of California San Diego School of Medicine, UNITED STATES

## Abstract

**Background:**

Trachomatous trichiasis significantly reduces vision and health related quality of life (QoL). Although trichiasis surgery is widely performed to treat trichiasis, there is little data on the effect of surgery on QoL. We measured the impact of trichiasis surgery on vision and health related QoL in a longitudinal study from Amhara Region, Ethiopia.

**Methodology/Principal Findings:**

We recruited 1000 adult participants with trichiasis (cases) and 200 comparison participants, matched to every fifth trichiasis case on age (+/- two years), sex and location. Vision-related quality of life (VRQoL) and health-related quality of life (HRQoL) were measured using the WHO/PBD-VF20 and WHOQOL-BREF questionnaires respectively, at enrolment and 12 months after enrolment. All trichiasis cases received free standard trichiasis surgery immediately after enrolment. The mean difference in QoL scores between enrolment and follow-up for cases and comparison participants, and the difference-in-differences by baseline trichiasis status was analysed using random effects linear regression, the later adjusted for age, sex and socioeconomic status. At 12-months follow-up, data was collected from 980 (98%) and 198 (98%) trichiasis cases and comparison participants respectively. At this follow-up visit, VRQoL and HRQoL scores of trichiasis cases improved substantially in all subscales and domains by 19.1–42.0 points (p<0.0001) and 4.7–17.2 points (p<0.0001), respectively. In contrast, among the comparison participants, there was no evidence of improvement in VRQoL and HRQoL domain scores during follow-up. The improvement in VRQoL and HRQoL in cases was independent of the presence of visual acuity improvement at 12 months.

**Conclusions/Significance:**

Trichiasis surgery substantially improves both VRQoL and HRQoL regardless of visual acuity change. Unprecedented effort is needed to scale-up trichiasis surgical programmes not only to prevent the risk of sight loss but also to improve overall wellbeing and health perception of affected individuals.

## Author Summary

We previously reported that Trachomatous Trichiasis (TT) has a profound impact on vision and heath related quality of life (QoL), even when vision is not impaired. The World Health Organization (WHO) recommends corrective eyelid surgery for trichiasis to reduce the risk of vision loss. However, trichiasis surgery may also improve overall wellbeing. There is very limited evidence on the long-term impact of trichiasis surgery on QoL. We measured vision and health-related quality of life of 1000 TT patients before and one year after receiving TT surgery and compared the QoL scores of these with the baseline and 1 year follow-up QoL score of 200 matched individuals who have never had trichiasis or trichiasis surgery. We found strong evidence that surgery substantially improves both vision and heath related QoL of TT case, even when there is no improvement in vision; while there was no evidence of improvement in the QoL of the trichiasis free participants. The results provide clear evidence that the benefit of trichiasis surgery goes beyond preventing the risk of blindness and improves the overall wellbeing and health perception of affected individuals, indicating the need to provide prompt surgical intervention for affected individuals.

## Introduction

Trachoma, an eye disease caused by *Chlamydia trachomatis*, is the leading infectious cause of blindness worldwide [1]. The infection can lead to progressive conjunctival scarring and subsequently trachomatous trichiasis, the in-turning of eyelashes. Trichiasis in turn can cause constant painful abrasion to the cornea, irreversible corneal opacification and ultimately visual impairment and blindness. Approximately 7.3 million people have untreated trichiasis, and 2.4 million people are visually impaired from trachoma worldwide [2,3].

Trachomatous trichiasis is a painful condition, which can have a major impact on the individual’s general health and wellbeing, even prior to the development of visual impairment [4]. Moreover, it may have major socioeconomic consequences for affected families and communities [4–6]. We have previously reported that trichiasis adversely impacts vision and health related quality of life (QoL), even before visual impairment develops [7]. Other studies have found trichiasis causes considerable functional and physical impairment, social withdrawal and exclusion, inability to work and earn an income [4,6,8,9].

The World Health Organization (WHO) recommends corrective eyelid surgery for trichiasis, to reduce the risk of sight loss [10]. Limited evidence suggests that the benefits of surgery may go beyond preventing vision loss and in fact help to restore the physical, social, psychological, environmental and economic wellbeing of individuals through improving vision and reducing pain and discomfort [8,11,12]. However, detailed empirical data on the impact of surgery on QoL is lacking. One longitudinal study from Ethiopia assessed the effect of trichiasis surgery on physical functioning of 411 trichiasis patients, six months after trichiasis surgery [9]. Another study, conducted in India, assessed HRQoL in 60 trachomatous entropion patients before and 1 month after trichiasis surgery and epilation [13]. No studies have measured the long-term overall effect that trichiasis surgery has on the different QoL domains and overall wellbeing.

The WHO has developed and validated several tools for measuring QoL. These include the WHO/PBD-VF20 which is designed to measure vision related quality of life (VRQoL); and the WHOQOL-BREF which measures general health related quality of life [14,15]. We have previously reported a case-control study, which used both of these tools to compare the QoL of individuals with trichiasis to healthy controls, and found substantial differences [7]. Here we report a longitudinal study of these same cases and controls (hereafter referred to as comparison participants), in which we explore the longer-term impact of trichiasis surgery on vision and health-related QoL.

## Methods

### Ethics Statement

This study was reviewed and approved by the National Health Research Ethics Review Committee of the Ethiopian Ministry of Science and Technology, the London School of Hygiene & Tropical Medicine (LSHTM) Ethics Committee, and Emory University Institutional Review Board. Written informed consent in Amharic was obtained prior to enrolment from participants. It was conducted in accordance with the Declaration of Helsinki. If the participant was unable to read and write, the information sheet and consent form were read to them and their consent recorded by thumbprint. Interviews were conducted privately, paper data were archived in a locked cabinet and electronic data were stored on a password-protected computer isolated from the Internet in a secured dedicated study office. Study participants with identified ocular problems were managed as per local protocol.

### Study Design and Participants

This longitudinal study was nested within a clinical trial of two alternative surgical treatments for trichiasis [16]. We recruited 1000 trichiasis cases into the trial, who were also enrolled into this QoL study. The pre-operative baseline data from this study have been previously reported in detail [7]. Cases were defined as individuals with one or more eyelashes touching the eyeball or with evidence of epilation in either or both eyes in association with tarsal conjunctival scarring. People with trichiasis from other causes, recurrent trichiasis and those aged <18 years were excluded. Trichiasis cases were identified mainly through community-based screening [17]. Recruitment was done in three districts of West Gojam Zone, Amhara Region, Ethiopia between February and May 2014. This area has one of the highest burdens of trachoma worldwide [18].

We also recruited 200 comparison participants. These were individuals without clinical evidence or a history of trichiasis (including epilation), who came from households without a family member with trichiasis or a history of trichiasis. Comparison participants were individually matched to every fifth trichiasis case by location, sex and age (+/- two years). The research team visited the sub-village (30–50 households) of the trichiasis case that required a matched control. A list of all potentially eligible people living in the sub-village of the case was compiled with the help of the sub-village administrator. One person was randomly selected from this list using a lottery method, given details of the study and invited to participate if eligible. If a selected individual refused or was ineligible, another was randomly selected from the list. When eligible comparison participants were not identified within the sub-village of the index case, recruitment was done in the nearest neighbouring sub-village, using the same procedures.

### Baseline Assessment

Data from trichiasis cases were collected at health facilities at the time of enrolment into the clinical trial, prior to trichiasis surgery. Data from the comparison participants were collected at their homes. Six trained Amharic speaking interviewers collected data from participants using a standardised questionnaire, including socio-demographic variables (age, sex, marital and literacy status), presence of other health problems in the last month and self-rated socioeconomic status (SES). For self-rated SES, participants were asked to rate the wealth of their households in relation to other households in their village by choosing one of the following options: (1) very poor, (2) poor, (3) average, (4) wealthy or (5) very wealthy [5].

#### Quality of life data

VRQoL was measured using the WHO/PBD-VF20 tool [14]. This contains 20 questions sub-divided into three subscales: visual symptom (3 questions), general functioning (12 questions) and psychosocial (4 questions). Each question has a five-point response option: one indicates the highest and five the lowest score. The psychosocial questions assess the frequency of experiencing a specific vision-related problem and the general functioning questions measure the difficulty associated with overall performance. This tool was translated into Amharic by two independent translators. The two translations were compared and differences were discussed and resolved to develop a single, final version.

HRQoL was measured using the WHOQOL-BREF [15]. This contains 26 questions, subdivided into four domains: physical health, psychological, social relationships and environment in the past four weeks [15,19,20]. The first two questions assess general QoL and health. Each item is scored on a five-point scale. We used the Amharic version provided by the WHO, which has been previously validated and used in Ethiopia [21–23].

#### Clinical data

Presenting LogMAR (Logarithm of the Minimum Angle of Resolution) visual acuity at two metres was measured using “PeekAcuity” software on a Smartphone in a dark room in both cases and controls [24]. We assessed contrast sensitivity with a prototype smartphone based test that presents the individual with calibrated grey scale spots against a white background, which they have to identify by touching the screen (www.peekvision.org). For cases, ophthalmic examination was conducted using a 2.5x binocular magnifying loupe and a bright torch. Clinical signs such as trichiasis severity and corneal opacity were graded using the Detailed WHO FPC Grading System [25].

### Surgical Intervention

Immediately after baseline data collection was completed, all cases received trichiasis surgical management. They were randomised to receive either the bilamellar or the posterior lamellar tarsal rotation, which were being compared in the clinical trial [16]. Both surgical procedures involve an incision through the scarred upper eyelid, parallel to and about 3mm above the lid margin, followed by outward rotation and suturing in the corrected position [26]. Six standardised trichiasis surgeons performed the surgery.

### Follow-up Assessment

Follow-up was conducted approximately one year after enrolment (minimum 10 and maximum 14 months), during the same season as the baseline. For cases, a reminder was sent to attend the 12-month follow-up. Follow-up data were collected for the majority of cases at a health facility. For cases who could not come to the health facility, data were collected during a home visit. Follow-up data were collected on comparison participants at their homes. Participants were interviewed using the same QoL tools at baseline, and clinical data were collected using the same procedures by the same interviewers and a clinical grader.

### Analysis

The sample size of 1000 cases and 200 comparison participants has 94% power to detect an effect size of about 0.27 (standardised mean difference (3/11)) with a Type I error of 5%. Data were double-entered into Access (Microsoft), cleaned in Epidata 3.1 and transferred to Stata 11 (StataCorp) for analysis. Analyses were restricted to participants with both baseline and follow-up data.

#### Quality of life data analyses

Items within each VRQoL sub-scale were summed to create a total sub-scale score which was then converted into a scaled value out of one hundred, using the formula: ([individual score—lowest possible score]/[highest possible score–lowest possible score]) x 100. Therefore, the person with the lowest possible VRQoL score would receive a scaled value of 0 and the person with the highest possible VRQoL score receives a scaled value of 100 [27]. Item scores were substituted by the mean of the subscale score in two cases with missing item scores in the visual symptom and psychosocial subscales separately.

The HRQoL data were analysed following the WHOQOL protocol [15,20]. Three negatively framed items were reversed into a positive frame so higher scores denote higher QoL. To generate domain scores, questions were grouped into their respective domains and their scores totalled. The mean score of all items included in the domain was calculated and then multiplied by four. These scores were then transformed to a 0 (lowest HRQoL) to 100 (highest HRQoL) scale [20]. Item scores were substituted by the mean of the domain score in cases with missing score in one item in the psychological domain and social domain, and in two items in the environment domain.

Mean change in VRQoL and HRQoL scores between baseline and follow-up was analysed using a random effect linear regression model separately for cases and comparison participants. This was repeated in stratified analysis by vision change at follow-up (better, same, worse) and analysis of variance was employed to test for significant difference in QoL score changes between those with better, same and worse vision. To test the null hypothesis that the difference in each QoL domain score between baseline and 12 month follow-up did not differ by case status, a difference-in-differences analysis was conducted using random effect linear regression with an interaction term included between visit (baseline and 12 month) and case status, adjusted for age, sex and self-rated SES [28]. The effect sizes for change between baseline and follow-up were estimated by dividing the mean score change for each QoL domain by the standard deviation of the baseline score separately for both cases and comparison participants. Effect sizes of <0.5, 0.5–0.79 and ≥0.8 were considered as small, moderate and large, respectively [29,30].

All comparisons of cases and comparison participants were adjusted for the matching variables (age and sex), and self-rated SES. Analyses of HRQoL analysis additionally adjusted for presence of other health problems during the previous four weeks, as these factors may confound the association between trachomatous trichiasis and HRQoL. Analysis was not adjusted for location as neighbourhood comparison participants were used. Logistic and linear regression methods adjusted for the matching variables were used for binary and continuous outcome analysis, respectively. P-values were obtained using likelihood ratio-tests.

Finally, we explored socio-economic and clinical predictors of change in VRQoL and HRQoL among cases between baseline and follow up using univariable and multivariable linear regression models. Tests for trend were undertaken in case of ordered categorical independent variables. Variables that were associated with the outcome on univariable analyses at a level of p<0.05 were included in the multivariable analysis and then those with p<0.2 were retained in the model. To adjust for multiple comparisons, we used the Benjamini and Hochberg method, assuming a false discovery rate (FDR) of 5%.

#### Clinical data analyses

To analyse vision and contrast sensitivity, the operated eye scores were used in unilateral cases; while the score in the better eye was used in comparison participants and cases with bilateral surgery. For visual acuities of counting fingers or less, LogMAR values were attributed as follows: counting fingers, 2.0; hand movements, 2.5; perception of light, 3.0; no perception of light, 3.5. The LogMAR visual acuity scores were then categorised based on the WHO classifications: Normal vision, >6/18; moderate visual impairment, <6/18 to ≥6/60; severe visual impairment, <6/60 to ≥3/60; and blind, <3/60. Contrast sensitivity scores were grouped into quartiles. The differences between baseline and follow-up scores were used to analyse longitudinal vision change. Participants were grouped into better vision (>0.1 LogMAR), same vision (-0.1 to 0.1 LogMAR) and worse vision (<-0.1 LogMAR) categories in relation to their baseline vision scores. Corneal opacity and trichiasis grading in the more affected eye was used in cases with bilateral surgery; while the operated eye grading was used in cases with unilateral surgery to test the association of these with QoL in trichiasis cases. Corneal opacity grading was categorised as either (i) no opacity/peripheral opacity or (ii) opacity involving the visual axis. Based on their baseline trichiasis severity, cases were categorised into Minor Trichiasis (<6 lashes or evidence of epilation in <1/3rd of the lashes) and Major Trichiasis (≥6 lashes or evidence of epilation in ≥1/3rd of the lashes). Recurrence in either of the operated eyes at 12 month was used to test its association with QoL domains.

## Results

### Demographic and Clinical Characteristics

At baseline 1000 trachomatous trichiasis cases and 200 comparison participants were recruited. At the 12-month follow-up, complete QoL data were collected from 980 (98%) cases and 198 (98%) comparison participants. Cases and comparison participants had a similar age distribution (mean 47.0 vs 45.7 years), but there were fewer females among the cases than among the comparison participants (76.4% vs 84.3%). This difference occurred by chance, as the randomly selected 200 trichiasis cases used to determine the matching characteristics had a higher proportion of females than the full group of 1000 trichiasis cases, Table 1. After adjusting analyses for age and gender, the trichiasis cases were significantly more likely to be illiterate, widowed or divorced, be from poorer households, and report other health problems in the past month than comparison participants, Table 1.

**Table 1:** Demographic and Clinical characteristics of participants seen at baseline and 12-months.

Variables		Cases		Comparison Participants		p-value
		n / 980	(%)	n / 198	(%)	
***Baseline***						
**Age, mean (SD)**		47.0 years	(14.8)	45.7 years	(13.2)	0.55
**Gender, female**		749	(76.4)	167	(84.3)	0.03
**Illiterate**		867	(88.5)	168	(84.8)	0.008
**Marital status**						
	Married	641	(65.4)	160	(80.8)	<0.0001^‡^
	Widowed	194	(19.8)	27	(13.6)	
	Divorced	114	(11.6)	9	(4.6)	
	Single	31	(3.2)	2	(1.0)	
**Job**						
	Farmer	823	(83.9)	166	(83.8)	0.03^‡^
	Employed/self employed	51	(5.2)	17	(8.6)	
	Daily labourer	45	(4.6)	4	(2.0)	
	No job	61	(6.2)	11	(5.6)	
Self-rated wealth*						
	Very wealthy/ Wealthy	32	(3.3)	30	(15.1)	<0.0001^†^
	Middle	442	(45.1)	133	(67.2)	
	Very Poor / Poor	506	(51.6)	35	(17.7)	
**Health problem**						
	No	618	(63.1)	172	(86.9)	<0.0001
	Yes	362	(36.9)	26	(13.1)	
**Visual Acuity** ^a^ **–best eye** ^¶^						
	Normal (≥6/18)	512	(52.2)	192	(97.0)	<0.0001^†^
	Moderate visual impairment (<6/18 to ≥6/60)	380	(38.8)	4	(2.0)	
	Severe visual impairment (<6/60 to ≥3/60)	31	(3.2)	1	(0.5)	
	Blind (<3/60)	57	(5.8)	1	(0.5)	
**Contrast sensitivity–best eye** ^¶^						
	1 (Best)	258	(26.3)	141	(71.2)	<0.0001^†^
	2	396	(40.4)	49	(24.7)	
	3	87	(8.9)	1	(0.5)	
	4 (Worst)	239	(24.4)	7	(3.5)	
***12-months follow-up***						
**Health problem**						
	No	553	(56.4)	161	(81.3)	<0.0001
	Yes	427	(43.6)	37	(18.7)	
**Visual Acuity** ^b^ **–best eye** ^¶^						
	Normal (≥6/18)	584	(59.7)	187	(94.4)	<0.0001^†^
	Moderate visual impairment (<6/18 to ≥6/60)	336	(34.4)	8	(4.0)	
	Severe visual impairment (<6/60 to ≥3/60)	18	(1.8)	1	(0.5)	
	Blind (<3/60)	40	(4.1)	2	(1.0)	
**Contrast sensitivity** ^b^ **–best eye** ^¶^						
	1 (Best)	*338*	(34.6)	145	(73.2)	<0.0001^†^
	2	182	(18.6)	23	(11.6)	
	3	244	(24.9)	13	(6.6)	
	4 (Worst)	214	(21.9)	17	(8.6)	

p-values are calculated using logistic regression and adjusted for age and gender, with the exception for age, which was calculated using linear regression.

^***‡***^ Combined p-value from likelihood ratio-test.

^†^ P-value for trend.

* We merged “very wealthy” and “wealthy”; and “very poor” and “poor” because of small numbers at these extremes of the distribution, to create three levels of socio-economic status measure to facilitate data modelling.

^**¶**^ Best eye vision and contrast sensitivity are presented for comparison participants and for cases with bilateral trichiasis surgery; while the operated eye visual acuity and contrast sensitivity are presented for cases with unilateral trichiasis surgery.

^a^ No data for 1 person.

^b^ Not possible to take visual acuity and contrast sensitivity in two participants.

At baseline almost all comparison participants (97%) had normal vision (≥6/18) compared with about half (52%) of the cases (in the operated eye). Trichiasis cases also had significantly lower contrast sensitivity score than the comparison participants at baseline (P<0.0001), and poorer visual acuity and contrast sensitivity scores at 12-months (Table 1). In contrast, at 12 months follow-up there was strong evidence of an improvement in visual acuity (mean LogMAR change: 0.08; 95%CI: 0.05 to 0.10) and contrast sensitivity scores (mean contrast sensitivity score change: 2.41; 95%CI: 1.48 to 3.35) among trichiasis cases. Moreover, among comparison participants there was a small but significant reduction in visual acuity (mean LogMAR change: -0.04; 95%CI: -0.07 to -0.02) and no evidence of a change in contrast sensitivity scores (mean contrast sensitivity score change: -0.14; 95%CI: -0.40 to 0.13, p = 0.31).

### Vision Related Quality Of Life

At baseline, trichiasis cases had substantially lower VRQoL scores than comparison participants in all four subscales (p<0.0001), Table 2. One year after trichiasis surgery the mean VRQoL score of cases had improved substantially in all subscales by 19.1 to 42.3 points (p<0.0001), with large effect sizes in the visual symptom (2.03), overall eyesight (1.57) and psychosocial (0.88) subscales and a moderate effect size in the general functioning (0.67) subscale. In contrast, there was no evidence of a difference between baseline and follow-up VRQoL scores in all subscales scores in the comparison participants and the effect sizes were very small and negative (-0.08 to -0.03), Table 2. The difference-in-differences analysis showed strong evidence that the improvement in mean VRQoL score from baseline to 12-month follow-up was greater for cases than comparison participants in all sub-scales (difference-in-differences score: 18.9 to 42.9 points, p<0.0001), Table 2.

**Table 2:** Comparison of mean VRQoL and HRQoL domain score changes and effect size between baseline and 12-months follow-up in trichiasis cases and comparison participants.

Variable		Baseline		Follow-up		Baseline v 12-month				
		*Mean*	*(95% CI)*	*Mean*	*(95% CI)*	*Difference*	*(95% CI)*	*P-value* ^b^	Effect size	*(95% CI)*
**VRQoL**										
Overall eyesight										
	Cases	46.3	(44.9–47.7)	81.3	(79.8–82.8)	35.0	(33.3–36.6)	<0.0001	1.57	(1.50–1.65)
	Comparison	95.4	(93.3–97.6)	94.8	(92.6–97.1)	-0.63	(-2.40–1.14)	0.48	-0.04	(-0.16–0.07)
		P<0.0001^a^		P<0.0001^a^		35.6 ^d^	(31.8–39.4)	<0.0001		
Visual symptom										
	Cases	46.2	(44.9–47.5)	88.5	(87.5–89.6)	42.3	(40.8–43.8)	<0.0001	2.03	(1.96–2.10)
	Comparison	97.6	(96.2–98.9)	97.0	(95.7–98.2)	-0.59	(-1.80–0.62)	0.34	-0.06	(-0.18–0.06)
		P<0.0001^a^		P<0.0001^a^		42.9 ^d^	(39.6–46.2)	<0.0001		
General functioning										
	Cases	74.2	(72.9–75.5)	93.3	(92.4–94.3)	19.1	(17.9–20.3)	<0.0001	0.67	(0.61–0.74)
	Comparison	97.7	(96.4–99.1)	97.9	(96.8–99.1)	0.21	(-0.87–1.29)	0.70	-0.08	(-0.21–0.05)
		P<0.0001^a^		P<0.0001^a^		18.9 ^d^	(16.2–21.6)	<0.0001		
Psychosocial										
	Cases	69.6	(67.9–71.2)	92.9	(91.9–94.0)	23.4	(21.7–25.0)	<0.0001	0.88	(0.81–0.94)
	Comparison	98.1	(96.8–99.5)	97.8	(96.4–99.3)	-0.25	(-1.33–0.82)	0.64	-0.03	(-0.14–0.09)
		P<0.0001^a^		P<0.0001^a^		23.6 ^d^	(19.9–27.3)	<0.0001		
**HRQoL**										
Physical health										
	Cases	47.8	(46.8–48.7)	64.9	(63.6–66.3)	17.1	(16.0–18.3)	<0.0001	1.11	(1.03–1.18)
	Comparison	80.1	(78.3–81.9)	76.5	(74.0–78.9)	-3.61	-5.48 –-1.74)	0.0002	-0.27	(-0.41–-0.13)
		P<0.0001^a^		P<0.0001^a^		20.8 ^d^	(18.1–23.4)	<0.0001		
Psychological										
	Cases	58.8	(58.0–59.6)	69.6	(68.6–70.6)	10.8	(9.78–11.8)	<0.0001	0.87	(0.79–0.95)
	Comparison	80.7	(79.2–82.2)	79.5	(77.9–81.1)	-1.18	(-2.72–0.37)	0.13	-0.11	(-0.25–0.03)
		P<0.0001^a^		P<0.0001^a^		12.0 ^d^	(9.58–14.4)	<0.0001		
Social										
	Cases	52.0	(50.6–53.4)	56.7	(55.3–58.1)	4.69	(3.29–6.10)	<0.0001	0.21	(0.14–0.27)
	Comparison	72.0	(69.6–74.4)	67.2	(64.6–69.7)	-4.88	(-7.20 –-2.57)	<0.0001	-0.29	(-0.42–-0.15)
		P<0.0001^a^		P<0.0001^a^		9.58 ^d^	(6.28–12.9)	<0.0001		
Environment										
	Cases	38.8	(38.2–39.5)	49.2	(48.3–50.1)	10.3	(9.43–11.2)	<0.0001	0.95	(0.87–1.03)
	Comparison	62.1	(60.6–63.7)	55.6	(53.8–57.4)	-6.55	(-8.45 –-4.64)	<0.0001	-0.58	(-0.75–-0.41)
		P<0.0001^a^		P<0.0001^a^		16.8 ^d^	(14.7–18.9)	<0.0001		

VRQoL = Vision Related Quality of Life

HRQoL = Health Related Quality of Life

^**a**^ p-values were calculated by linear regression and adjusted for age, gender and self-rated wealth (and other health problem in past month for HRQoL domains) comparing cases and comparison participants QoL scores at baseline and follow-up.

^**b**^ p-values from random effect linear regression adjusted for age, gender and self-rated wealth (and other health problem in past month for HRQoL domains) comparing QoL change between baseline and follow-up separately for case and comparison participants

^**d**^ Difference-in-differences analysis conducted using random effect linear regression with interaction term included between visit (baseline and 12 month) and case/ comparison status and adjusted for age, gender and self-rated wealth (and other health problem in past month for HRQoL domains)

Using the Benjamini and Hochberg method, only tests with a p-value below 0.0003 have a False Discovery Rate of <5%.

Similar results were seen when analyses were stratified by level of visual acuity change at 12-months, Table 3. VRQoL score among trichiasis cases improved significantly in all subscales, independent of visual changes over 12-months, while the VRQoL scores of comparison participants remained the same (Table 3). The largest VRQoL change was observed in trichiasis cases with visual improvement, Table 3. In addition, among trichiasis cases, VRQoL improved in all subscales independent of postoperative recurrent trichiasis, although the improvements in the overall eyesight was significantly less in those with recurrent trichiasis than their counterparts. (30.8 vs 35.7; p = 0.04).

**Table 3:** Comparison of mean change in VRQoL and HRQoL domain scores between baseline and 12-months in trichiasis cases and comparison participants, stratified by the change in vision between baseline and 12-months (Better, Same and Worse).

Variable		Vision Better			Vision Same			Vision Worse			ANOVA
		*Mean change*	*(95% CI)*	*P-value* ^a^	*Mean change*	*(95% CI)*	*P-value* ^a^	*Mean change*	*(95% CI)*	*P-value* ^a^	P-value ^c^
**VRQoL**											
Overall eyesight											
	Cases	40.1	(37.5–42.7)	<0.0001	33.5	(30.8–36.1)	<0.0001	28.4	(24.9–31.9)	<0.0001	<0.0001
	Comparison participants	-2.00	(-8.90–4.89)	0.57	-0.70	(-2.48–1.08)	0.44	0.0	(-3.73–3.73)	1.000	0.83
	Difference-in-Differences ^b^	42.1	(31.8–52.4)	<0.0001	34.2	(29.1–39.2)	<0.0001	28.4	(21.7–35.0)	<0.0001	
Visual symptom											
	Cases	44.7	(42.4–47.1)	<0.0001	41.9	(39.7–44.2)	<0.0001	38.4	(35.2–41.6)	<0.0001	0.0056
	Comparison participants	0.33	(-5.20–5.87)	0.91	-0.62	(-1.68–0.43)	0.25	-0.88	(-3.36–1.59)	0.48	0.99
	Difference-in-Differences ^b^	44.4	(35.1–53.6)	<0.0001	45.6	(38.3–46.9)	<0.0001	39.3	(33.3–45.3)	<0.0001	
General functioning											
	Cases	22.5	(20.5–24.4)	<0.0001	17.1	(15.3–18.9)	<0.0001	16.4	(13.9–19.0)	<0.0001	0.033
	Comparison participants	1.75	(-5.39–8.89)	0.63	-0.43	(-1.15–0.30)	0.25	0.66	-0.74–2.07)	0.36	0.59
	Difference-in-Differences ^b^	20.7	(12.8–28.7)	<0.0001	17.5	(14.2–20.8)	<0.0001	15.8	(11.2–20.4)	<0.0001	
Psychosocial											
	Cases	25.6	(22.9–28.3)	<0.0001	22.3	(19.7–24.8)	<0.0001	21.1	(17.6–24.6)	<0.0001	0.12
	Comparison participants	-1.00	(-6.99–4.99)	0.74	-0.17	(-0.77–0.42)	0.56	-0.09	(-2.22–2.03)	0.93	0.94
	Difference-in-Differences ^b^	26.6	(16.0–37.1)	<0.0001	22.4	(17.6–27.3)	<0.0001	21.2	(14.8–27.5)	<0.0001	
**HRQoL**											
Physical health											
	Cases	17.1	(15.3–18.9)	<0.0001	18.1	(16.4–19.8)	<0.0001	15.8	(13.3–18.3)	<0.0001	0.62
	Comparison participants	0.14	(-5.58–5.86)	0.96	-3.14	(-5.22–1.05)	0.0032	-5.79	(-9.67–-1.90)	0.0035	0.016
	Difference-in-Differences ^b^	17.0	(9.67–24.2)	<0.0001	21.2	(17.8–24.7)	<0.0001	21.6	(16.6–26.5)	<0.0001	
Psychological											
	Cases	10.9	(9.27–12.5)	<0.0001	11.6	(9.93–13.3)	<0.0001	9.36	(7.17–11.5)	<0.0001	0.80
	Comparison participants	1.00	(-4.08–6.08)	0.70	-1.13	(-2.95–0.69)	0.22	-2.08	(-5.11–0.94)	0.18	0.06
	Difference-in-Differences ^**e**^	9.90	(3.39–16.4)	0.0029	12.8	(9.42–16.1)	<0.0001	11.4	(7.16–15.7)	<0.0001	
Social											
	Cases	5.23	(3.02–7.44)	<0.0001	4.76	(2.53–7.00)	<0.0001	3.91	(0.76–7.07)	0.015	0.80
	Comparison participants	0.33	(-6.30–7.00)	0.92	-5.84	(-8.92–-2.76)	0.0002	-5.30	(-9.40–-1.23)	0.011	0.06
	Difference-in-Differences ^b^	4.90	(-3.95–13.7)	0.28	10.6	(6.11–15.1)	<0.0001	9.22	(3.09–15.3)	0.0032	
Environment											
	Cases	9.17	(7.82–10.5)	<0.0001	11.8	(10.4–13.2)	<0.0001	9.90	(8.03–11.8)	<0.0001	0.12
	Comparison participants	-8.87	(-15.4–-2.35)	0.0077	-6.25	(-8.80–-3.70)	<0.0001	-6.15	(-9.27–-3.04)	0.0001	0.71
	Difference-in-Differences ^b^	18.0	(12.5–23.6)	<0.0001	18.0	(15.0–21.0)	<0.0001	16.0	(12.3–19.8)	<0.0001	

To analyse vision change, best eye vision and contrast sensitivity were used for comparison participants and for cases with bilateral trichiasis surgery; while the operated eye visual acuity and contrast sensitivity were used for cases with unilateral trichiasis surgery. “Mean” refers to the mean difference between baseline and 12month follow-up QoL scores. VRQoL = Vision Related Quality of Life. HRQoL = Health Related Quality of Life.

^**a**^ p-values from random effect linear regression adjusted for age, gender and self-rated wealth (and other health problem in past month for HRQoL domains) comparing QoL change between baseline and follow-up within each vision change group, separately for case and comparison participants

^**b**^ Difference in differences analysis conducted within each vision change group using random effect linear regression with interaction term included between visit (baseline and 12 month) and case/ comparison status and adjusted for age, gender and self-rated wealth (and other health problem in past month for HRQoL domains)

^**c**^ p-value from ANOVA (controlling for age, gender, self-rated wealth for the VRQoL subscales; and controlling for age, gender, self-rated wealth and other health problem in the past month for the HRQoL domains) to test for a difference of mean change for each domain, between better, same and worse vision groups.

Using the Benjamini and Hochberg method, only tests with a p-value below 0.034have a False Discovery Rate of <5%.

#### Health related quality of life

At baseline, trichiasis cases had substantially lower HRQoL scores than comparison participants in all four domains (p<0.0001), Table 2. One year after trichiasis surgery, the HRQoL scores of trichiasis cases had significantly improved in all four domains (mean improvement: 4.7 to 17.2 points; p<0.0001). In contrast, the HRQoL scores for comparison participants had reduced at 12-months in all four domains. The effect sizes were large for trichiasis cases in the physical health (1.11) psychological (0.87) and environment (0.95) domains and small for the social domain (0.21). In contrast, the effect sizes for comparison participants were negative. The difference-in-differences analysis showed that the change in mean HRQoL score was significantly greater for cases than comparison participants for all four domains (9.58 to 20.8 points, p<0.0001) Table 2.

Results stratified by visual acuity change at 12-months showed that there was significant improvement in HRQoL scores of trichiasis cases in all domains, independent of visual change over 12-months Table 3, but no improvement among the comparison participants Table 3.

#### Determinants of quality of life change in trichiasis cases

The relationship between socio-demographic and clinical factors and change in VRQoL and HRQoL among trichiasis cases 12 months after surgery are presented in Table 4. In multivariable analysis, greater improvements in all VRQoL subscales were independently associated with longer trichiasis duration, central corneal opacity at baseline and improved vision at 12-months. In addition, cases with bilateral trichiasis surgery had greater gains in visual symptom and general functioning subscales scores than those with monocular surgery. Cases with poor contrast sensitivity scores at baseline had significantly greater improvement in general functioning scores than their counterparts. Younger individuals had greater improvement in the visual symptom and psychosocial domain scores; while older individuals had greater improvement in the general functioning subscale score.

**Table 4:** Determinants of VRQoL and HRQoL change between baseline and 12-months after trichiasis surgery.

Variable		VRQoL						HRQoL							
		Visual symptom		General functioning		Psychosocial		Physical		Psychological		Social		Environment	
		*Mean change*	*(95% CI)*	*Mean change*	*(95% CI)*	*Mean change*	*(95% CI)*	*Mean change*	*(95% CI)*	*Mean change*	*(95% CI)*	*Mean change*	*(95% CI)*	*Mean change*	*(95% CI)*
**Age Groups (years)**															
	≤ 29	43.2	(39.2–47.1)	12.0	(8.1–15.9)	25.1	(20.1–30.0)	21.5	(18.6–24.4)	14.6	(11.9–17.2)	5.1	(0.67–9.6)	14.4	(11.7–17.2)
	30–39	44.9	(42.1–47.8)	11.7	(9.1–14.3)	23.9	(20.4–27.4)	22.2	(19.6–24.8)	14.7	(12.4–17.1)	8.5	(5.5–11.4)	13.2	(11.2–15.1)
	40–49	43.3	(40.4–46.3)	14.6	(12.0–17.2)	25.7	(22.3–29.1)	17.1	(14.7–19.5)	11.4	(9.5–13.4)	3.4	(0.55–6.3)	10.2	(8.4–12.0)
	50–59	43.7	(40.5–46.9)	14.9	(11.8–18.0)	25.5	(22.0–29.0)	14.8	(12.3–17.2)	9.8	(7.6–12.1)	5.9	(2.8–9.0)	8.3	(6.5–10.1)
	60–69	36.3	(32.1–40.5)	13.9	(10.0–17.7)	17.7	(13.4–21.9)	13.2	(10.4–16.0)	7.4	(4.6–10.3)	4.2	(0.40–7.9)	8.4	(6.2–10.5)
	≥ 70	40.2	(34.6–45.8)	21.4	(16.5–26.2)	19.0	(13.2–24.8)	13.8	(10.4–17.1)	4.3	(0.72–7.9)	-2.5	(-6.6–1.6)	7.5	(4.9–10.0)
	p-value ^a^ ^†^	0.008		0.003		0.01		<0.0001		<0.0001		0.01		<0.0001	
	p-value ^b^ ^†^	0.0001		-		0.0001		<0.0001		<0.0001		0.03		<0.0001	
**Gender**															
	Male	40.7	(37.8–43.5)	11.7	(9.2–14.3)	19.8	(16.6–23.0)	18.7	(16.5–21.0)	14.8	(12.8–16.8)	4.4	(1.1–7.6)	12.7	(11.0–14.4)
	Female	42.8	(41.1–44.5)	15.1	(13.5–16.7)	24.5	(22.6–26.4)	16.7	(15.4–18.0)	9.6	(8.4–10.8)	4.8	(3.2–6.3)	9.6	(8.6–10.6)
	p-value ^a^	0.22		0.04		0.02		0.13		<0.0001		0.80		0.003	
	p-value ^b^	-		0.16		-		-		<0.0001		-		<0.0001	
**Trichiasis severity**															
	Minor trichiasis (<6 lashes)	40.9	(39.0–42.9)	13.0	(11.2–14.9)	21.7	(19.5–23.9)	17.6	(16.1–19.2)	11.9	(10.5–13.2)	4.2	(2.3–6.1)	10.5	(9.3–11.7)
	Major trichiasis (≥6 lashes)	43.8	(41.7–46.0)	15.8	(13.8–17.7)	25.2	(22.8–27.6)	16.6	(15.0–18.3)	9.6	(8.1–11.1)	5.3	(3.2–7.3)	10.0	(8.8–11.3)
	p-value ^a^	0.05		0.04		0.03		0.39		0.03		0.46		0.58	
	p-value ^b^	-		-		-		-		-		-		-	
**Trichiasis duration in years**															
	<2	32.4	(29.1–35.8)	8.2	(5.1–11.3)	14.4	(10.8–18.8)	16.6	(14.1–19.1)	9.0	(6.4–11.6)	5.5	(2.0–9.0)	8.9	(6.6–11.2)
	2–4	43.5	(41.0–46.1)	14.9	(12.5–17.2)	24.9	(21.9–27.9)	17.3	(15.2–19.4)	11.2	(9.5–13.0)	3.7	(1.0–6.5)	11.7	(10.2–13.2)
	5–9	44.9	(42.1–47.7)	15.4	(12.7–18.0)	25.1	(21.7–27.5)	17.6	(15.3–19.9)	12.6	(10.5–14.9)	6.3	(3.3–9.3)	9.9	(8.1–11.8)
	10–15	43.4	(39.3–47.6)	13.6	(9.8–17.3)	25.8	(21.3–30.2)	15.8	(12.8–18.9)	9.0	(6.3–11.7)	3.0	(0.02–6.0)	9.3	(7.1–11.4)
	≥15	45.9	(42.0–49.9)	19.3	(15.3–22.8)	25.6	(21.5–29.8)	17.9	(15.0–20.8)	10.7	(7.8–13.5)	4.5	(1.1–8.0)	10.6	(8.4–12.8)
	p-value ^a^ ^†^	<0.0001		0.0002		0.0007		0.79		0.76		0.76		0.97	
	p-value ^b^ ^†^	<0.0001		0.01		0.0001		-		-		-		-	
**Baseline corneal opacity**															
	No/peripheral opacity	40.7	(39.0–42.5)	11.7	(10.2–13.3)	21.4	(19.5–23.3)	16.9	(15.6–18.3)	11.4	(10.1–12.6)	6.0	(4.3–7.8)	10.5	(9.4–11.5)
	Opacity involving the visual axis	45.2	(42.6–47.8)	19.1	(16.6–21.6)	27.0	(24.1–30.0)	17.5	(15.5–19.5)	9.7	(7.9–11.6)	2.2	(-0.09–4.4)	10.0	(8.6–11.4)
	p-value ^a^	0.004		<0.0001		0.001		0.64		0.14		0.01		0.60	
	p-value ^b^	0.02		0.001		0.0004		-		-		0.04		-	
**Baseline presenting VA**															
	Normal (≥6/18)	42.8	(41.0–44.7)	12.0	(10.4–13.5)	22.2	(20.1–24.3)	18.5	(17.0–20.0)	12.7	(11.4–14.0)	5.8	(3.8–7.8)	12.3	(11.1–13.4)
	MVI (<6/18 to ≥6/60)	41.7	(39.1–44.3)	15.6	(13.2–18.0)	24.0	(21.3–26.7)	16.0	(14.2–17.9)	9.0	(7.2–10.7)	3.6	(1.4–5.9)	8.0	(6.6–9.5)
	SVI (<6/60 to ≥3/60)	44.3	(35.9–52.8)	23.2	(14.2–32.3)	26.8	(16.6–37.0)	11.4	(4.58–18.2)	5.8	(-0.28–11.8)	-3.8	(-9.7–2.2)	10.2	(5.5–14.9)
	Blind (<3/60)	40.9	(33.7–48.1)	22.3	(15.1–29.5)	28.2	(19.1–37.3)	15.6	(10.7–20.4)	8.6	(3.5–13.7)	6.7	(1.1–12.2)	7.5	(4.5–10.5)
	p-value ^a^ ^†^	0.55		<0.0001		0.05		0.02		0.0008		0.28		0.0001	
	p-value ^b^ ^†^	-		-		-		-		-		-		-	
**Baseline contrast sensitivity**															
	4 (Best score)	41.1	(38.5–43.7)	9.4	(7.1–11.7)	19.6	(16.6–22.5)	20.2	(18.1–22.2)	14.4	(12.5–16.3)	7.7	(5.0–10.3)	14.3	(12.5–16.1)
	3	44.1	(41.8–46.4)	14.4	(12.4–16.4)	25.5	(22.9–28.0)	16.9	(15.2–18.7)	10.3	(8.7–11.8)	3.1	(0.9–5.4)	9.3	(8.0–10.6)
	2	41.2	(36.4–45.9)	13.8	(9.4–18.2)	23.3	(17.9–28.7)	16.3	(12.2–20.3)	10.2	(6.8–13.5)	2.5	(-2.7–7.7)	9.2	(6.5–11.9)
	1 (Worst score)	41.1	(37.9–44.3)	19.6	(16.4–22.8)	23.9	(20.4–27.5)	14.5	(12.2–16.9)	8.0	(5.7–10.4)	4.8	(2.1–7.6)	8.0	(6.3–9.7)
	p-value ^a^ ^†^	0.65		<0.0001		0.16		0.0007		0.0001		0.25		<0.0001	
	p-value ^b^ ^†^	-		<0.0001		-		-		0.02		-		-	
**Surgery**															
	Unilateral	40.3	(38.6–42.0)	13.2	(11.6–14.8)	22.0	(20.2–23.9)	17.0	(15.7–18.3)	10.9	(9.7–12.1)	4.6	(3.0–6.2)	9.6	(8.6–10.6)
	Bilateral	48.1	(45.4–50.7)	17.5	(14.9–20.2)	27.2	(18.9–28.1)	17.6	(15.3–19.9)	10.5	(8.4–12.6)	5.0	(2.1–7.9)	12.2	(10.5–14.0)
	p-value ^a^ ^†^	<0.0001		0.006		0.0070		0.65		0.73		0.81		0.01	
	p-value ^b^ ^†^	0.0007		0.01		-		-		-		-		0.007	
**Surgical procedure**															
	PLTR	43.1	(41.0–45.1)	15.3	(13.4–17.2)	24.1	(21.7–26.4)	16.9	(15.3–18.4)	11.2	(9.8–12.7)	4.9	(2.9–6.9)	10.0	(8.8–11.3)
	BLTR	41.5	(39.5–43.6)	13.3	(11.4–15.2)	22.7	(20.3–25.0)	17.4	(15.8–19.0)	10.4	(8.8–11.9)	4.5	(2.5–6.5)	10.6	(9.4–11.7)
	p-value ^a^ ^†^	0.31		0.15		0.40		0.63		0.40		0.77		0.56	
	p-value ^b^ ^†^	-		-		-		-		-		-		-	
**Vision change at 12 month**															
	Better	44.7	(42.4–47.1)	16.8	(14.6–19.1)	25.6	(22.9–28.2)	17.1	(15.3–18.9)	10.9	(9.3–12.5)	5.2	(3.0–7.4)	9.2	(7.8–10.5)
	Same	41.9	(39.7–44.2)	12.9	(10.8–14.9)	22.3	(19.7–24.8)	18.1	(16.4–19.8)	11.6	(9.9–13.3)	4.8	(2.5–7.0)	11.8	(10.4–13.2)
	Worse	38.4	(35.2–41.6)	12.0	(9.2–14.9)	21.1	(17.6–24.6)	15.8	(13.3–18.3)	9.4	(7.2–11.5)	3.9	(0.8–7.1)	9.9	(8.0–11.8)
	p-value ^a^ ^†^	0.001		0.004		0.03		0.53		0.39		0.50		0.28	
	p-value ^b^ ^†^	0.001		0.01		0.05		-		-		-		-	

^a^ P-values from univariable analysis.

^b^ P-values from multivariable analysis adjusted. All p-values are calculated using linear regression. For ordinal exposures with three or more categories the ^†^p-values are calculated for trend. Using the Benjamini and Hochberg method, only tests with a p-value below 0.020 have a False Discovery Rate of <5%. Variables with univariable p<0.05 were included in the multivariable model, then those with p>0.2 were excluded (dashed line) from the final model after likelihood ratio- test. VRQoL = Vision related Quality of Life.

For the HRQoL, young age was independently associated with larger improvements in all domains; while males had greater improvement in psychological and environment domain scores than females (Table 4). Cases with better baseline contrast sensitivity; no/peripheral baseline corneal opacity and bilateral surgery had larger psychological, social and environment domain scores improvements than their counterparts’ respectively. The type of surgical procedure (PLTR or BLTR) was not associated with differences in either change in vision or health related QoL (Table 4). Hence, all QoL change results are presented combining all operated Trachomatous Trichiasis cases.

## Discussion

Trichiasis surgery is primarily performed to reduce the risk of sight loss [10]. Between 2004 and 2013, about 1.2 million operations were performed worldwide, and currently around 200,000 trichiasis cases are treated annually [31]. However, despite this activity, there is very little longitudinal information on the impact of trichiasis surgery on quality of life. Cross-sectional studies, including the baseline report of this study, have shown that trichiasis is associated with a reduced quality of life and causes marked disability [6,7,13]. In this study we measured the vision and health related QoL of trichiasis patients before and one year after trichiasis surgery using standard WHO assessment tools. We also compared these outcomes with the QoL scores of individuals who have never had trichiasis, matched by age, gender and location to the cases. We found strong evidence that trichiasis surgery substantially improves both VRQoL and HRQoL 12-months after treatment, even when there is no improvement in vision; while the scores for the comparison participants were largely unchanged.

### Vision Related Quality of Life

For VRQoL, the largest improvement was seen in the visual symptom subscale indicating the major effect that surgery has on relieving pain and discomfort from trichiasis. Trichiasis surgery is also shown to significantly improve visual acuity and contrast sensitivity, which might be related to elimination of the photophobia and tears and restoration of the corneal epithelium as result of the removal of the trichiatic lashes [8,11]. Larger gains in VRQoL were seen in those with improved vision. However, substantial improvement in VRQoL also occurred in patients without visual acuity improvement or even in those with deteriorating vision. This strongly supports the view that trichiasis surgery should be performed not only to save vision but also to treat other debilitating symptoms and improve the VRQoL of affected individuals. In contrast, the comparison participants had no improvement in visual acuity, contrast sensitivity or VRQoL 12-months after enrolment.

There has been no previous longitudinal study quantifying the effect of trichiasis surgery on VRQoL. However, our findings are consistent with a retrospective qualitative study of 13 women with trichiasis in Niger, in which most participants reported that trichiasis surgery markedly improved their quality of life in association with a complete disappearance of the painful physical symptoms [6]. Interestingly, despite a fundamental difference between cataract and trichiasis in the amount of visual loss, the effect size for the improvement in overall eyesight between our study and a study of the impact of cataract surgery in Kenya was comparable (1.6 vs 1.5) and there were large effect sizes (>0.80) in the psychosocial subscales in both studies [30].

### Health Related Quality of Life

Among trichiasis cases, there was marked improvement in HRQoL one year after trichiasis surgery, independent of change in vision. The largest improvement was seen in the physical health domain indicating that trichiasis surgery considerably improves work capacity and ability to function without pain. Similar findings have been reported in the Niger qualitative study [6]. In contrast, there was either reduction or no change in the HRQoL of comparison participants one year after enrolment.

The results of our study were consistent with the only longitudinal study on HRQoL and trichiasis, conducted in India, which assessed HRQoL in patients before and one month after trichiasis surgery for trachomatous entropion (n = 41) and 15 days after epilation for minor trichiasis cases (n = 19). In the Indian study, HRQoL significantly improved after treatment of patients with normal and poor vision in the physical health, psychological and environment domains, while there was no significant improvement in the social domain. Consistent with our study, the greatest and least improvements were seen in the physical health (mean 21.3 to 23.1 points) and social domains (2.0 to 3.3 points), respectively. The social domain is a composite of social support and personal relationships, which may be less altered by trichiasis surgery.

The QoL improvement in our study was larger in the three HRQoL domains except the physical domain, than reported in the Indian study. These two studies have the following differences that might explain the differences in HRQoL gains. Firstly, most of the cases (67%) in the Indian study had entropion without trichiasis while all had trachomatous trichiasis in our study. Secondly, 19 cases with minor trichiasis received epilation (rather than surgery) while everyone in our study underwent trichiasis surgery. Thirdly, there was less than one month of follow-up in the Indian study during which the surgical wound healing process might influence QoL results [13].

### Predictors of Change in Quality of Life of Trichiasis Cases

Longer trichiasis duration and central corneal opacity at baseline predict larger VRQoL gains in all subscales. This suggests that cases with severe disease probably benefit more in terms of improved VRQoL following trichiasis surgery. The results also suggest that bilateral cases benefit more from bilateral surgery to restore their functioning. Older trichiasis cases had greater improvement in the general functioning subscale than younger cases, while younger cases showed greater improvement in the psychosocial scores. Older individuals had more difficulty in general functioning in relation to distance and near vision difficulties at baseline, hence should benefit more from trichiasis surgery in improving their vision and thereby participation in day to day activities. Younger cases may be more likely to be embarrassed, worry about losing eyesight and hesitate to participate in social activities due to their trichiasis than older cases, which may be the reason for younger trichiasis cases to having greater improvement in the psychosocial subscale than older people one year after trichiasis surgery.

### Strengths and Limitations

This is the first large longitudinal study to measure VRQoL and HRQoL change after trichiasis surgery using validated WHO tools. The same interviewers collected data at both baseline and follow-up to ensure questionnaires were administered in a standard way at baseline and follow-up. The study used comparison participants. Absence of significant improvement in QoL among the comparison participants compared to the cases lends weight to the view that the positive QoL change observed is attributable to trichiasis surgery. The study has a number of limitations. The interviewers were not masked in the trichiasis status of the participants and we cannot rule out the possibility of response bias with cases providing a positive answer to satisfy the interviewers regardless of real change or improvement. To limit these, participants were asked to provide honest answers and reassured that their answers would not affect their treatment in any way. In addition, knowledge of trichiasis status by the interviewers might possibly introduced bias in the outcome assessment. Although the improved QoL probably is largely related to vision improvement, less pain and irritation after the surgery, it is difficult to rule out the possibility that the extent of positive change observed among cases might have been related to the effect of just receiving a health intervention rather than solely attributed to the effect of trichiasis surgery. This possibly would explain the 28.4 score improvement in the overall eyesight subscale in those with vision deterioration one year after trichiasis surgery.

### Conclusions

Overall this study demonstrated that trichiasis surgery substantially improves both vision and health related QoL regardless of the visual acuity improvement, suggesting that the effect of trichiasis surgery goes beyond preventing the risk of blindness and improves the overall wellbeing and health perception of affected individuals. Unprecedented effort is needed to scale-up trichiasis surgical programmes and provide prompt surgical intervention to improve overall wellbeing of affected individuals.

## Supporting Information

S1 Checklist STROBE checklist. (DOCX)
